# Growth in children with biliary atresia before and after liver transplantation: a retrospective analysis

**DOI:** 10.3389/fped.2025.1715315

**Published:** 2026-01-12

**Authors:** Ye Wang, Pingping Zhang, Ji Qi, Yanting Guo

**Affiliations:** Department of Paediatrics, Tianjin First Central Hospital, Tianjin, China

**Keywords:** biliary atresia, catch-up growth, children, liver transplantation, nutritional status

## Abstract

**Background:**

Little is known about the long-term survival and growth outcomes of children following liver transplantation in China. Therefore, we investigated the long-term survival and growth status of children with biliary atresia (BA) before and after liver transplantation and attempted to identify specific influencing factors.

**Methods:**

We included children who underwent liver transplantation for BA at Tianjin First Central Hospital between January 2014 and December 2018. Clinical data, indicators of liver function, growth data, and ascites status were collected for analysis from all patients. Postoperative complications were systematically assessed using a combination of clinical, laboratory, histological, and imaging evaluations. Complications were categorized and graded according to the Clavien–Dindo classification system.

**Results:**

This study included a total of 93 children under 2 years of age who underwent liver transplantation for BA. No significant increase in height was observed six months post-transplantation when compared to pre-transplantation (*P* = 0.126). A significant increase in the height-for-age *Z*-score occurred by year 2 (*P* = 0.001). Preoperative growth was negatively correlated with growth improvement at 2- and 5-years after transplantation (*P* < 0.001, r = −0.674 and r = −0.774, respectively). The occurrence of biliary complications was significantly associated with impaired catch-up growth post-transplantation (*P* = 0.008). The weight-for-age *Z*-score increased significantly over the first two years post-transplantation when compared with that before transplantation (*P* < 0.001); subsequently, this parameter plateaued and exhibited only minimal changes.

**Conclusions:**

Growth retardation is common in children with BA prior to liver transplantation, with catch-up growth commencing six months post-surgery and peaking after two years. Children with more severe preoperative growth delays exhibited faster postoperative growth. Preoperative growth status exhibited only minimal impact on early liver function recovery post-transplantation.

## 1 Introduction

Biliary atresia (BA), a condition of unknown origin, is characterized by obstruction of the intrahepatic and extrahepatic bile ducts and can lead to bile stasis, progressive liver fibrosis, and cirrhosis, thus posing a life-threatening risk to affected children (1). Without timely intervention, progressive cirrhosis can result in death before a child has reached 2 years-of-age (2). The incidence of BA is known to vary across races and regions. Globally, BA affects approximately 1 in every 10,000 live births and accounts for approximately 30% of all cases of neonatal cholestasis in pediatric patients (3). Liver transplantation is the only effective treatment for end-stage BA, and in China, the increasing accessibility of liver transplantation has shifted focus to postoperative concerns, such as quality-of-life, growth, development, the risks associated with immunosuppression, and the prevention of graft fibrosis. Growth and development are important indicators of the overall health of children with chronic diseases. Children with end-stage liver disease frequently experience malnutrition and growth retardation (4, 5). However, following successful liver transplantation, many children experience catch-up growth as liver functionality improves, and over time, some children may even achieve a normal height and weight for their age (6).

Congenital BA is one of the primary indications for pediatric liver transplantation, and with the advancement of surgical techniques and improvements in immunosuppressive therapy in China, an increasing number of children undergoing liver transplantation are achieving long-term survival. In this study, long-term survival was defined as a postoperative survival time of at least three years with stable graft function, characterized by a total bilirubin ≤2-fold higher than the upper limit of normal (ULN) and alanine aminotransferase (ALT) ≤ 2 × ULN, without the need for secondary transplantation. Growth normalization is a critical parameter for evaluating the success of treatments for chronic diseases in the pediatric population. Although several studies have reported growth and developmental outcomes in Chinese pediatric patients following liver transplantation for biliary atresia (5, 7, 8), these investigations were generally limited by small sample sizes, short follow-up durations (mostly <2 years), and a lack of standardized anthropometric indicators such as *Z*-scores. As such, long-term survival and comprehensive growth assessments in this population remain insufficiently characterized.

Therefore, in this retrospective study, we analyzed the growth status of children with BA before and after liver transplantation over a five-year follow-up period. We also aimed to investigate the potential influencing factors affecting postoperative growth outcomes. Although survival rates were not the primary outcome of this study, we used long-term survival to characterize the postoperative status of patients with functioning grafts. In addition, we systematically evaluated how preoperative growth and nutritional status, assessed by height-for-age *Z*-scores (HAZ) and weight-for-age *Z*-scores (WAZ), along with serum albumin, bilirubin, and vitamin D levels, affected postoperative liver function recovery and catch-up growth. Growth improvement was quantified as the change in HAZ from baseline to two years post-transplantation, and liver function recovery was defined by the normalization of key liver enzymes and bilirubin within 30 days post-surgery. Our findings aim to provide valuable clinical insights for optimizing preoperative evaluation and postoperative management in this population.

## 2 Methods

### 2.1 Study design and population

This retrospective study included a total of 93 children <2 years-of-age who underwent liver transplantation for BA at our hospital between January 2014 and December 2018 and received regular follow-up. The inclusion criteria were as follows: Patients under 2 years-of-age who underwent liver transplantation at our hospital for biliary atresia and received regular postoperative follow-up. The exclusion criteria were as follows: patients who died within 5 years postoperatively or were completely lost to follow-up within 2 years postoperatively. The study was reviewed and approved by the Ethics Committee of Tianjin First Central Hospital (Approval Reference: 2021NO52KY). All procedures were conducted in accordance with the ethical standards of the institutional and/or national research committee and with the 1964 Declaration of Helsinki and its later amendments. Written informed consent was obtained from all participants prior to data collection.

### 2.2 Data collection

All patient information was obtained from the electronic medical records system of our hospital. The key observation indicators included clinical data, indicators of liver function, growth data and ascites status. Clinical data included sex, age at the time of transplantation, and whether Kasai surgery was performed prior to liver transplantation. Indicators of liver function included preoperative and postoperative liver function parameters, including alanine aminotransferase, aspartate aminotransferase, albumin, and total bilirubin, which were measured using an automated biochemical analyzer (cobas 701, Roche Diagnostics, Basel, Switzerland), as well as the time to postoperative liver function recovery (defined as the point at which transaminase and bilirubin levels normalized). Growth data included height and weight, measured preoperatively and at 6 months, one year, two years, three years, four years, and five years post-transplantation. Body length/height and weight were measured using standardized pediatric measuring devices, including an infant height and weight measuring instrument (TH996C, Leaguer Technology Co., Ltd., Shenzhen, China) and an electronic height and weight scale (RCS-200, Jiangsu Suhong Medical Instrument Co., Ltd., Jiangsu, China). The corresponding *Z*-scores were calculated. The WAZ and height-for-age *Z*-score (HAZ) were computed according to child growth standards published by the World Health Organization (Geneva) (WHO) (9), using Equation (1).

(1) Z-score=(measuredvalue–median)/standarddeviation

According to the WHO child growth standards, a HAZ score < −2 indicates growth retardation, whereas a WAZ score < −2 indicates an underweight condition (9). Ascites status was assessed clinically and confirmed by abdominal ultrasound, performed using color Doppler ultrasound systems (ACUSON OXANA 3, Siemens Healthineers, Erlangen, Germany; LOGIQ E9, GE Healthcare, Chicago, IL, USA) with convex transducers (frequency range: 1–6 MHz). The severity of ascites was categorized as minimal (a small amount of fluid collection that was detectable only on ultrasound), moderate (clinically detectable fluid with mild abdominal distension), and significant (gross abdominal distension with fluid shifting on physical examination).

### 2.3 Assessment and management of postoperative complications

Postoperative complications were systematically assessed using a combination of clinical, laboratory, histological, and imaging evaluations. During hospitalization, patients underwent daily clinical examinations and weekly laboratory tests, including liver function parameters, complete blood counts, and tacrolimus trough levels, which were measured using a chemiluminescent immunoassay system (ARCHITECT i1000SR, Abbott Laboratories, Abbott Park, IL, USA). Scheduled protocol liver biopsies were performed at 1-, 3-, and 12-months post-transplantation, and abdominal ultrasound was conducted when clinically indicated.

Complications were categorized and graded according to the Clavien–Dindo classification system (10, 11). Management strategies were implemented based on the type and severity of the complication. For acute rejection, we administered glucocorticoid pulse therapy as follows: 10 mg/kg on Day 1, 5 mg/kg on Days 0–1, 4 mg/kg on Days 1–2, 3 mg/kg on Days 2–3, 2 mg/kg on Days 3–4, and 2 mg/kg thereafter. We maintained tacrolimus blood levels between 15 and 20 ng/mL. The subsequent immunosuppressive regimen was as follows: glucocorticoids 2 mg/kg/day, with tacrolimus blood levels maintained at 5–10 ng/mL (10, 11); refractory cases received basiliximab or mycophenolate mofetil. Biliary leakage was managed with endoscopic retrograde cholangiopancreatography (ERCP) and stenting or surgical repair. Lymphatic fistulas were initially managed conservatively (e.g., a low-fat diet or compression) and surgically ligated if persistent. Hepatic artery thrombosis was treated with anticoagulation therapy [heparin; target activated partial thromboplastin time (APTT) 60–80 s], followed by thrombolysis or thrombectomy, if necessary.

Infectious complications were treated based on microbiological findings. Bacterial infections were treated with broad-spectrum antibiotics (e.g., carbapenems and vancomycin), fungal infections with anti-fungals (e.g., voriconazole and caspofungin), and cytomegalovirus (CMV) with valganciclovir and intravenous immunoglobulin (IVIG). Diaphragmatic hernias were corrected surgically. Late complications included biliary strictures, treated with endoscopic balloon dilation and/or stenting; intrahepatic bile duct stenosis, treated with percutaneous transhepatic cholangial drainage (PTCD) or surgical reconstruction; portal vein stenosis, managed by observation or angioplasty; and post-transplant lymphoproliferative disorder (PTLD), treated by immunosuppression reduction and rituximab or chemotherapy.

All interventions were guided by multidisciplinary consultation to ensure timely diagnosis and optimal treatment.

### 2.4 Statistical analysis

The Shapiro–Wilk test was used to assess the normality of continuous variables prior to statistical comparisons. Continuous variables are presented as mean (± standard deviation) or median (interquartile range), depending on normality, whereas categorical data are expressed as frequency. Group comparisons were conducted according to the following predefined subgroups: (1) growth status: preoperative growth retardation (HAZ < −2) vs. normal growth (HAZ ≥ −2); (2) biliary complications: presence vs. absence of postoperative biliary strictures or infections; (3) liver function recovery: early recovery (≤10 days) vs. delayed recovery (>10 days). For comparisons between two groups, independent-sample *t*-tests or Mann–Whitney *U*-tests were used, depending on normality. For comparisons among three or more groups, analysis of variance (ANOVA) or Kruskal–Wallis tests were applied accordingly. Correlations between continuous variables were assessed using Pearson's correlation coefficient (for normally distributed data) or Spearman's rank correlation (for non-parametric data). Longitudinal changes in HAZ and WAZ scores at different time points (preoperative, 6 months, 2 years, and 5 years post-transplant) were analyzed using repeated-measures ANOVA. To identify independent predictors of catch-up growth (defined as ΔHAZ ≥ 0.25), multivariate logistic regression analyses were performed, adjusting for potential confounders including age, sex, and the presence of ascites. Data were analyzed using SPSS version 26.0 software (IBM Corp., Armonk, NY, USA), and *P* < 0.05 was considered statistically significant.

## 3 Results

### 3.1 Patient demographics and treatment status

In total, 93 patients (54 females and 39 males) were included in this study, with a median age of 7.0 months (4–23 months). Of these, 36 patients (38.7%) exhibited ascites at the time of transplantation, including 18 with minimal ascites, 12 with moderate ascites, and six with significant ascites. Table 1 shows a summary of basic patient characteristics.

**Table 1:** Baseline clinical characteristics of 93 children with biliary atresia undergoing liver transplantation.

Item	Subcategory	Value
Sex	Male	39 (41.9%)
	Female	54 (58.1%)
Age at transplantation (months)	Median (IQR)	7.0 (4.0–23.0)
Weight (kg)	Median (IQR)	7.0 (4.7–12.0)
Height (cm)	Median (IQR)	64.0 (57.0–76.0)
Kasai surgery	Yes	43 (46.2%)
	No	50 (53.8%)
Donor type	Brain-dead donor	24 (25.8%)
	Living donor	69 (74.2%)
Type of donor liver	Split liver	77 (82.8%)
	Whole liver	16 (17.2%)
Ascites status	Minimal^a^	18 (19.4%)
	Moderate^b^	12 (12.9%)
	Significant^c^	6 (6.5%)
	Total with ascites	36 (38.7%)
Liver function indicators	AST^d^ (U/L)	331.1 (80.0–1,810.7)
	ALT^e^ (U/L)	194.7 (18.6–1,824.9)
	GGT^f^ (U/L)	291.0 (24.3–3,341.0)
	TBIL^g^ (*μ*mol/L)	248.9 (19.2–734.2)
	ALB^h^ (g/L)	33.2 (24.6–44.0)

^a^ Minimal ascites: Detectable only on ultrasound.

^b^ Moderate ascites: Clinically detectable with mild abdominal distension.

^c^ Significant ascites: Gross distension with fluid shifting on physical exam.

^d^ AST: aspartate aminotransferase.

^e^ ALT: alanine aminotransferase.

^f^ GGT: gamma-glutamyl transferase.

^g^ TBIL: total bilirubin.

^h^ ALB: albumin.

Data are presented as the *n* (%) or median (interquartile range).

Normality tested by Shapiro–Wilk; Mann–Whitney *U*-test used for non-normal variables.

The initial postoperative immunosuppressive regimen consisted of tacrolimus (target trough level: 5–10 ng/mL) in combination with prednisolone (initial dose: 2 mg/kg/day, tapered weekly). All patients discontinued prednisolone within the first year after surgery, and 92% (*n* = 86) discontinued prednisolone within six months, with a mean discontinuation time of 4.2 ± 1.8 months. Liver function recovery was monitored by daily measurements of serum bilirubin and transaminase levels until normalization. The median time to normalization of serum bilirubin and transaminase levels was 10 days (interquartile range: 5–68 days), with a mean duration of 23.74 ± 12.56 days. The postoperative complications experienced by patients are presented in Table 2.

**Table 2:** Postoperative complications according to postoperative growth status of the 93 children.

Complication		Total cases (*n* = 93)	Cases with normal growth^a^ (*n* = 58, 62.4%)	Cases without normal growth (*n* = 35, 37.6%)
Early complications	Graft rejection	8 (8.6%)	3 (37.5%)	5 (62.5%)
	Biliary leakage	2 (2.2%)	1 (50.0%)	1 (50.0%)
	Lymphatic fistula	2 (2.2%)	1 (50.0%)	1 (50.0%)
	Hepatic artery thrombosis	1 (1.1%)	0 (0.0%)	1 (100.0%)
	Infect	2 (2.2%)	0 (0.0%)	2 (100.0%)
	Diaphragmatic hernia	1 (1.1%)	0 (0.0%)	1 (100.0%)
Late complications	Biliary anastomotic stricture	11 (11.8%)	2 (18.2%)	9 (81.8%)
	Intrahepatic bile duct stenosis	2 (2.2%)	0 (0.0%)	2 (100.0%)
	Portal vein stenosis	4 (4.3%)	1 (25.0%)	3 (75.0%)
	Posttransplant lymphoproliferative disorder	4 (4.3%)	0 (0.0%)	4 (100.0%)

^a^ Normal growth: Defined as a HAZ score ≥ −2 at two years post-transplantation.

### 3.2 The most significant improvement in height occurred during the first two years after liver transplantation

**Figure 1:**
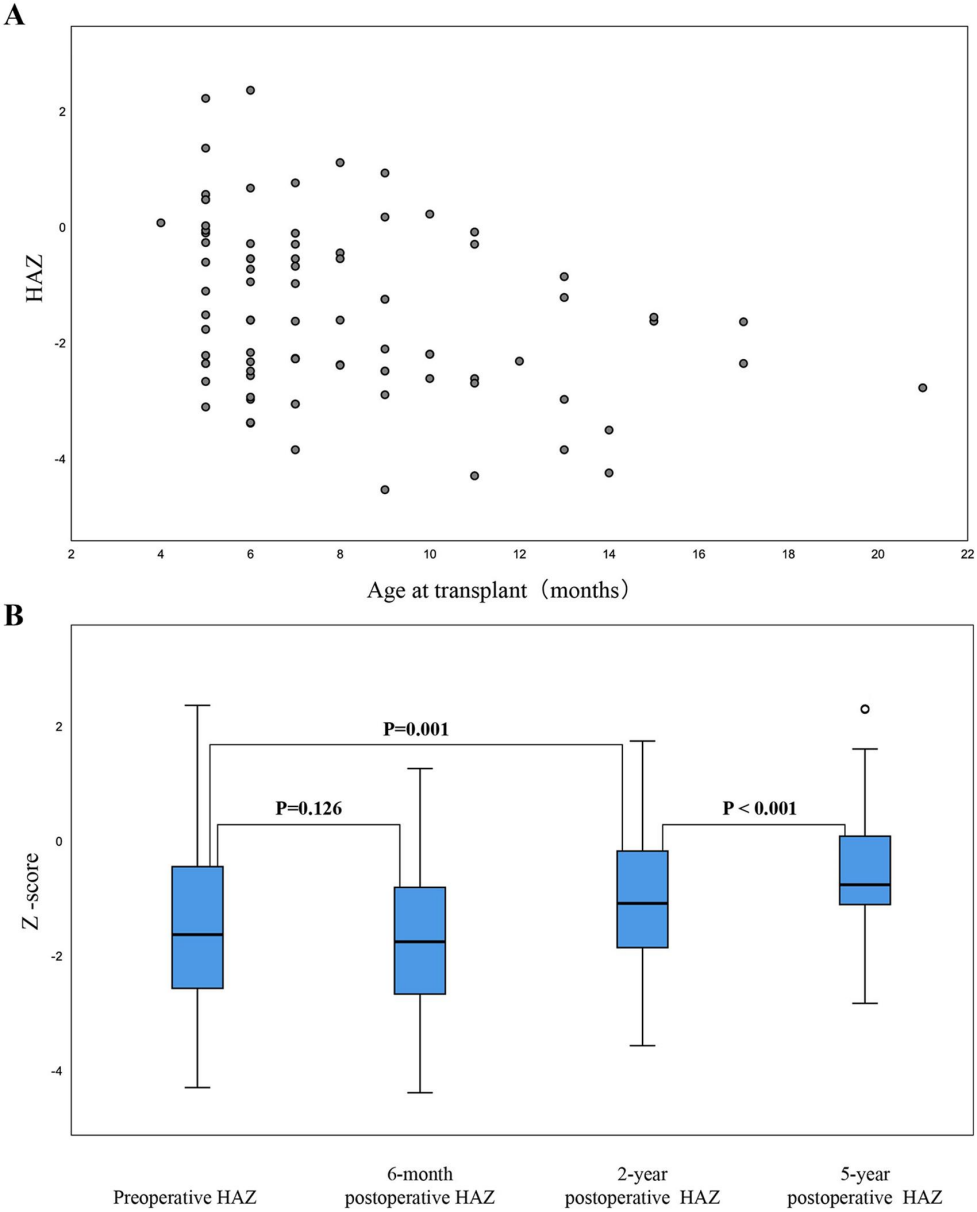
Distribution and longitudinal comparison of height-for-age *Z*-scores before and after liver transplantation. **(A)** Distribution of height-for-age *Z*-scores at the time of liver transplantation; **(B)** Longitudinal comparison of height-for-age *Z*-scores before and after liver transplantation.

Prior to surgery, 45 children exhibited growth retardation, while 17 were underweight (Table 3). The median preoperative HAZ score was −1.65 (−4.56 to 2.34). Of the total cohort of patients, 45 children had a HAZ score < −2. Figure 1A shows the distribution of HAZ scores at the time of liver transplantation.

**Table 3:** Preoperative growth status of the 93 children with biliary atresia.

Sex	No.^a^ of Cases	Growth Retardation^b^	Underweight^c^
Male	39	19	10
Female	54	26	7
Totab	93	45	17

^a^ No.: number.

^b^ Growth Retardation: HAZ score < −2.

^c^ Underweight: WAZ score < −2.

Two years after transplantation, the median HAZ score had improved to −1.55 (−3.59 to 1.72), and had improved further five years after transplantation to −0.56 (−2.85 to 2.28). Overall, the mean HAZ score exhibited significant improvement over time (*P* < 0.05). Six months post-transplantation, no substantial height increase was observed (mean difference: 1.06, *P* = 0.126; Figure 1B). Conversely, two years post-transplantation, ANOVA revealed a significant increase in the mean HAZ score when compared with preoperative scores (mean difference: 1.47, *P* = 0.001; Figure 1B). For most patients, the greatest improvement in growth occurred within the first two years after transplantation.

Ninety-three patients were followed up for two years after transplantation. Of these, 20 children still exhibited a HAZ score < −2, whereas 58 children showed an increased HAZ score (0.06–4.21). Of these 58 children with increased height, 41 exhibited an annual increase in the HAZ score of at least 0.25, reaching the criteria for catch-up growth (10).

Owing to differences in family compliance, only 83 patients were followed-up for five years post-transplantation. Of these, four children had a HAZ score < −2, whereas 55 children showed an increased HAZ score (0.03–3.21). Furthermore, the mean HAZ score increased significantly between two- and five-years post-transplantation (mean difference: 0.91, *P* < 0.05; Figure 1B).

**Figure 2:**
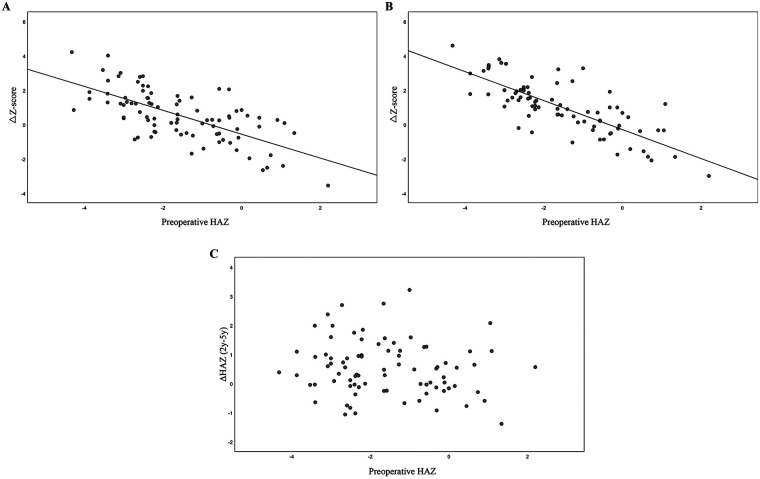
Analysis of the correlation between preoperative height status and catch-up growth at post-transplantation. **(A)** Negative correlation between preoperative height status and catch-up growth at 2 years post-transplantation; **(B)** Negative correlation between preoperative height status and catch-up growth at 5 years post-transplantation. **(C)** Lack of correlation between preoperative HAZ and growth progression from 2 to 5 years post-transplantation.

Subsequently, growth status was compared between the 0–2-year and 0–5-year post-transplantation periods. We identified a negative correlation between preoperative growth status and growth improvement, represented by ΔZ (ΔZ = HAZ score at two or five years – HAZ score at transplant), at both two years (r = −0.674, *P* < 0.001; Figure 2A) and five years (r = −0.774, *P* < 0.001; Figure 2B). Conversely, no significant correlation was detected between preoperative growth retardation and growth status during the 2–5 years post-transplantation period (*P* = 0.169; Figure 2C). This further indicated that the greatest improvement in growth status occurred within the first two years following liver transplantation.

### 3.3 The most significant improvement in weight occurred during the first two years after liver transplantation

The median WAZ score at the time of transplantation was −1.24 (−3.28–1.63). However, this may not have accurately reflected the nutritional status of the group because several patients had ascites prior to transplantation; consequently, for each child, the recorded weight did not reflect their dry weight. At two years post-transplantation, the median WAZ score had improved to 0.14 (−2.17 to 2.35), and by five years, the median WAZ score was 0.08 (−1.05 to 1.69).

**Figure 3:**
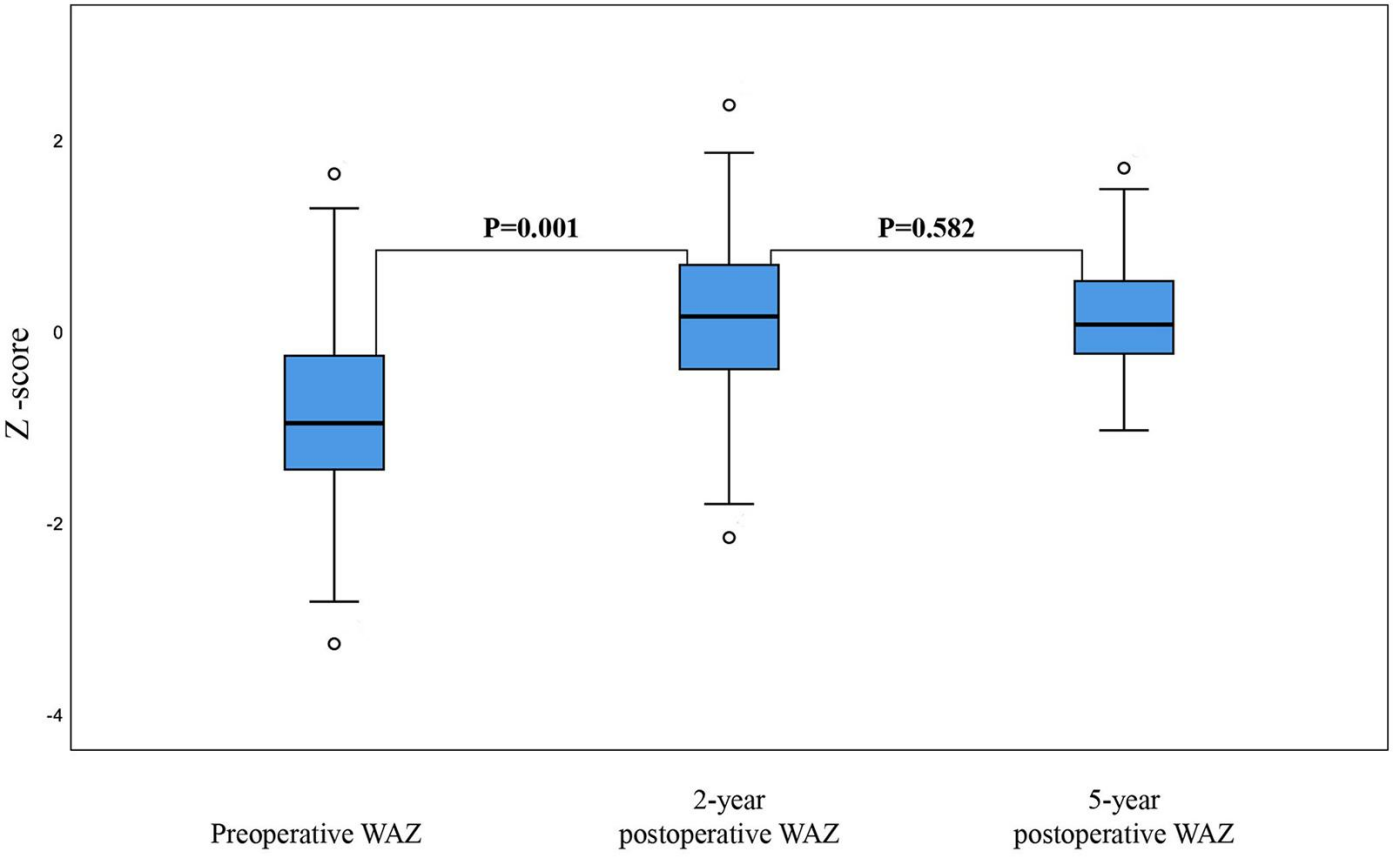
Changes in weight-for-age *Z*-scores before and after liver transplantation.

Similarly, when compared with the mean WAZ score pre-transplantation, the mean WAZ score increased significantly within the first two years post-transplantation (mean difference: 0.1, *P* = 0.001; Figure 3). However, the mean WAZ score plateaued after two years, with no significant change in the mean WAZ score when compared between two- and five-years post-transplantation (mean difference: 0.075, *P* = 0.582; Figure 3).

### 3.4 Growth status had no impact on postoperative outcomes

**Figure 4:**
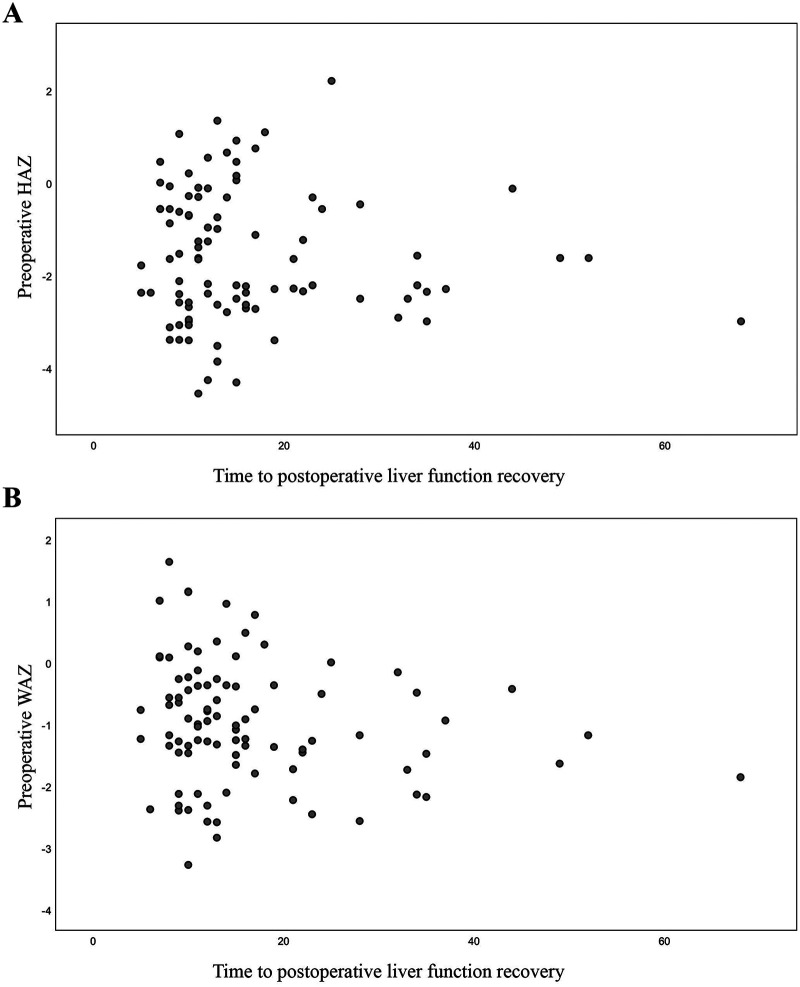
Correlation between preoperative HAZ and WAZ and time to postoperative liver function recovery. **(A)** Correlation between preoperative HAZ and time to postoperative liver function recovery; **(B)** Correlation between preoperative WAZ and time to postoperative liver function recovery.

Using a HAZ score of < −2 to define growth retardation, the 93 patients were divided into a normal growth group and a growth retardation group based on their preoperative height. In the growth retardation subgroup (*n* = 45), the median time to the postoperative recovery of liver function was 13 days (5–68 days), with a mean of 16.62 days. Pearson's correlation analysis found no significant correlation between the preoperative HAZ score and the time to postoperative liver function recovery (R = −0.065, *P* = 0.534; Figure 4A). Similarly, no significant correlation was detected between the preoperative WAZ score and the time to postoperative liver function recovery (R = −0.184, *P* = 0.078; Figure 4B).

### 3.5 The primary factors affecting postoperative catch-up growth were biliary complications

Our analysis included 45 children with growth retardation prior to surgery; of these, 45 were followed up for two years after surgery. As shown in Table 4, 29 of the 45 children (64.4%) achieved catch-up growth (defined as an increase in HAZ score ≥0.25 at two years post-transplantation), whereas 16 children (35.6%) did not achieve this threshold. A comparison of the demographic and clinical characteristics between the two groups revealed no significant differences in terms of sex distribution (*P* = 0.612), median age at transplantation (*P* = 0.212), preoperative height (HAZ) (*P* = 0.864), or total bilirubin levels (*P* = 0.503). Furthermore, there were no statistically significant differences in the proportion of children who underwent Kasai surgery prior to transplantation (*P* = 0.818) or in the median time to postoperative liver function recovery (*P* = 0.211). However, the proportion of biliary complications was significantly higher in the group that did not exhibit catch-up growth (56.2%) when compared to the group that did exhibit catch-up growth (10.3%, *P* = 0.008). Furthermore, the mean change in HAZ score (*Δ*HAZ) at two years post-transplantation was significantly greater in the group exhibiting catch-up growth (1.82 ± 0.54) than that in the group that did not attain catch-up growth (0.89 ± 0.41, *P* < 0.001), indicating a strong association between biliary complications and impaired postoperative growth (Table 4).

**Table 4:** Analysis of factors influencing postoperative catch-up growth.

Variables	Total (*n* = 45)	Group 1: Catch-up Growth^a^ (*n* = 29, 64.4%)	Group 2: No Catch-up Growth (*n* = 16, 35.6%)	*P*-value	Statistic^c^
Sex, *n* (%)					
Female	22 (48.9%)	15 (51.7%)	7 (43.8%)	0.612	*χ*^2^ = 0.26
Male	23 (51.1%)	14 (48.3%)	9 (56.2%)		
Age at transplantation (months), Median (Q1, Q3)	7.5 (6, 10)	7 (6, 9)	10 (6.5, 11.5)	0.212	Z = 1.555
Height (HAZ), Median (Q1, Q3)	−2.64 (−3.12, −2.36)	−2.64 (−3.08, −2.39)	−2.64 (−3.64, −2.27)	0.864	Z = 0.029
TBIL (μmol/L), Mean ± SD	226.02 ± 123.18	235.66 ± 114.74	208.66 ± 139.57	0.503	T = 0.457
Kasai surgery, *n* (%)				0.818	χ^2^ = 0.053
No	22 (48.9%)	13 (44.8%)	9 (56.2%)		
Yes	23 (51.1%)	16 (55.2%)	7 (43.8%)		
Biliary complications, *n* (%)				0.008	Fisher's test
Yes	12 (26.7%)	3 (10.3%)	9 (56.2%)		
No	33 (73.3%)	26 (89.7%)	7 (43.8%)		
Time to liver function recovery (days), Median (Q1, Q3)	13 (10, 19)	13 (9, 17.5)	13 (12, 21)	0.211	Z = 1.564
Doses of glucocorticoids(g), Median (Q1, Q3)	2.8 (5.0, 6.6)	2.1 (1.0, 4.1)	5.2 (4.9, 6.6)	0.136	Z = −1.492
^b^*Δ*HAZ (2 years post-transplant), Mean ± SD	1.47 ± 0.62	1.82 ± 0.54	0.89 ± 0.41	< 0.001	T = 6.123

a Catch-up growth: Defined as an increase in HAZ score ≥0.25 at 2 years post-transplantation.

b *Δ*HAZ: Change in HAZ score from pre-transplantation to two years post-transplantation.

c Statistical tests: χ^2^ for categorical variables, *t*-test for continuous variables, Fisher's exact test for small sample sizes.

## 4 Discussion

### 4.1 Patterns of post-transplantation catch-up growth

Our analysis revealed that growth retardation was prevalent among children with end-stage liver disease caused by BA. Moreover, catch-up growth, a rapid growth phase following a period of growth suppression in which growth rate surpasses what is typical for a child's age or developmental stage, was commonly observed in these patients following liver transplantation. This mechanism helps children return to their original growth trajectory prior to the onset of stunted growth, provided the conditions for growth are favourable (12).

Previous studies have investigated the growth patterns of children before and after liver transplantation (6, 13–18). To mitigate the confounding effects of age at the time of transplantation, we included children younger than two years-of-age as our study participants. The prevalence of growth retardation (a HAZ score < −2) among children with BA undergoing liver transplantation was 48.4% (45/93), whereas the proportion of children who were underweight (a WAZ score < −2) was 18.3% (17/93). At the time of transplantation, the mean HAZ and WAZ scores were −1.37 ± 1.43 and −1.22 ± 0.98, respectively, thus indicating that the growth status of children was generally within the normal range, with a mean value that was only slightly inferior to their healthy peers of the same age.

In the present study, no significant increase in height was observed within the first six months after transplantation. Instead, catch-up growth occurred during the following 18 months. This initial growth delay was attributed to the use of corticosteroids (5); as the steroid dosage was reduced, catch-up growth became more apparent. Similarly, multiple studies have reported that children with preoperative growth retardation experience catch-up growth within 1–2 years after surgery; this trend is particularly prevalent in malnourished children and those undergoing transplantation before two years-of-age (16, 17, 19–21). These findings were consistent with those derived from our present analysis. Furthermore, the greatest improvement in *Z*-scores occurred during the first two years after transplantation, while growth subsequently slowed between years two and five.

A small proportion of patients with preoperative growth retardation still exhibited impaired growth (HAZ < −2) at five years post-transplantation, consistent with previous reports (16, 17, 20, 22). A previous long-term study on post-transplantation growth previously suggested that height and weight normalize within 10–15 years after liver transplantation (20). Conversely, data from a large North American cohort revealed that 23% of children experienced impaired linear growth within 10 years post-transplantation (19). Therefore, future research should incorporate longer follow-up periods.

The rate of catch-up growth following liver transplantation is associated with the degree of preoperative growth retardation. In our study, we observed that children with more severe preoperative growth retardation exhibited faster postoperative growth, a pattern that was consistent with the findings of other studies (20, 22).

Similar to the observed pattern of height growth, weight gain peaked within the first two years post-transplantation. However, unlike height, the WAZ score did not increase significantly between years two and five. We hypothesize that certain factors may have influenced preoperative weight measurements, such as hypo-proteinemia and ascites, which are typical of end-stage liver disease. The presence of ascites, particularly in its significant form, likely influenced these measurements, as the recorded weight of each child did not reflect their dry weight.

Using the 2005 growth charts for children from nine Chinese cities as a reference ensured that our findings were more representative of the local population. While the WHO growth standards provide a global benchmark, regional variations in growth patterns necessitate the use of population-specific references. For example, Chinese children typically exhibit slow growth during the first two years of life when compared to the WHO standards; this may have influenced the interpretation of growth retardation and inferior weight in this cohort (23, 24).

### 4.2 Association between preoperative growth status and the postoperative recovery of liver function

Multiple factors are known to contribute to growth retardation in children with end-stage liver disease. For example, in the final stages of liver disease, liver dysfunction leads to impaired bile secretion and a reduction in the synthesis and storage of carbohydrates, proteins, and fats, thus causing malnutrition and stunted growth (25). Liver dysfunction, along with malnutrition due to the loss of muscle and fat, increases basal energy expenditure, thus placing children in a hypermetabolic state. Pierro et al. previously reported that children with BA may have energy requirements that are 30%–40% higher than normal (26). Additional factors can further reduce energy intake and exacerbate a child's energy deficit, including gastric compression caused by hepatosplenomegaly and ascites, poor appetite, and malabsorption. Furthermore, endocrine disruptions can make a substantial contribution to growth impairment in children with chronic liver disease. Specifically, disruption of the growth hormone insulin-like growth factor 1 axis due to liver dysfunction is an important factor that can induce growth retardation in children with chronic liver disease. Vitamin D, a steroid hormone, plays a significant biological role in bone health and the metabolism of calcium and phosphorus. Vitamin D deficiency and hepatic osteodystrophy are frequently reported in children with end-stage liver disease and are both associated with bone pain, growth retardation, and fractures, as noted in previous studies (27). Previous studies have also shown that low levels of 25-(OH) D(25-hydroxyvitamin D) are associated with reduced bone density among children with chronic liver disease (28).

Our analysis revealed that preoperative growth status did not exert significant effects on the early postoperative recovery of liver function. Although children with growth retardation exhibited a slightly higher complication rate post-transplantation than those with normal growth, the difference in complication rates was likely to have been influenced by multiple factors, including recurrent preoperative infections, the quality of the donor liver, surgical methods, and fluctuations in the levels of immunosuppressants, which were not comprehensively accounted for in our study. Therefore, the observed differences in complication rates cannot be solely attributed to growth status. Similarly, a previous Chinese study of 131 children with BA who underwent liver transplantation found no correlation between growth status and the postoperative recovery of liver function, long-term survival, or the incidence of complications (29).

### 4.3 Factors influencing growth and development in children after liver transplantation

A multitude of factors can influence the growth and development of children following liver transplantation; these can be classified into pre-transplantation and post-transplantation factors. Pre-transplantation factors include age at transplantation, the type of primary disease, the time from diagnosis of the primary disease to transplantation, the HAZ score at transplantation, and tumor presence/type. Post-transplantation factors include graft dysfunction/chronic rejection, the total dose of steroids used in the first-year post-transplantation, the history of hepatic vein stenosis, post-transplantation lymphoproliferative disorder, donor age, feeding status, and the type of primary disease (14).

Recent studies have also demonstrated that children who received whole liver transplantation exhibited better growth than those who received split liver transplantation, that biliary complications can influence height development, and that children with longer postoperative hospital stays exhibit reduced height and body mass index (BMI) percentiles (30). Overall, our analysis showed that biliary complications exerted significant impact on catch-up growth in children with preoperative growth retardation during the first two years after transplantation, while gender, age at transplantation, preoperative height, and liver function recovery time had no significant impact. However, other factors discussed in the literature were not included in our analysis, including corticosteroid exposure, donor liver type (whole vs. split), postoperative nutritional interventions, and graft function. This limitation primarily originates from the homogeneity of our study population in certain aspects. For instance, all patients discontinued corticosteroids within the first-year post-transplantation (92% within six months), with minimal variability in the duration of steroid exposure. This uniformity precluded meaningful analysis of the impact of corticosteroids on growth outcomes, although previous studies have established its association with delayed catch-up growth (5, 22). Similarly, donor liver type (whole vs. split) was not included as a variable due to the predominance of split liver transplants (77/93 cases) in our cohort. While existing evidence suggests that whole liver transplantation may yield better growth outcomes (30), our limited sample size of whole liver recipients (*n* = 16) restricted comparative analysis.

Our study indicates that the total amount of hormone administered has no significant effect on catch-up growth during the two-year postoperative period, which contradicts findings from other existing studies. A possible reason is that all patients discontinued corticosteroids within the first year post-transplant (92% within 6 months), with minimal variation in steroid exposure duration. This consistency precludes meaningful analysis of corticosteroids' impact on growth outcomes, despite prior studies demonstrating their association with delayed catch-up growth (5, 20). Similarly, donor liver type (whole vs. split) was not included as a variable because split-liver transplants predominated in this cohort (77/93 cases). Although existing evidence suggests whole-liver transplantation may yield superior growth outcomes (28), the limited sample size of whole-liver recipients (*n* = 16) precluded comparative analysis.

In addition, preoperative nutritional interventions, such as fortified feeding or parenteral nutrition, were not systematically recorded in this retrospective study, despite their potential role in modifying postoperative growth trajectories. A recent meta-analysis highlighted the lack of robust evidence supporting preoperative nutritional optimization in pediatric liver transplantation (31), thus aligning with our decision to focus on objectively measurable variables such as *Z*-scores and complications.

### 4.4 Preoperative nutritional management recommendations

Many studies have been conducted over the years in various countries to address the nutritional needs of individuals following liver transplantation, and different recommendations have been proposed. Of these, the recommendations on nutritional support for children with chronic liver disease jointly published by the North American Society for Pediatric Gastroenterology, Hepatology, and Nutrition and the European Society for Pediatric Gastroenterology, Hepatology, and Nutrition in 2019 are relatively comprehensive (32). However, one recent meta-analysis suggested that there is currently insufficient evidence to show that preoperative nutritional intervention exerts significant benefits for the short- or long-term prognosis of children after transplantation (31).

### 4.5 Limitations

There are some limitations to this study that need to be considered. These include a small sample size, a lack of data relating to the dry weight of children, and the insufficient assessment of children's growth, development, and nutritional status during the preoperative evaluation. Nevertheless, our results show that during the preoperative period for liver transplantation, it is important to provide comprehensive assessment and individualized nutritional support to address various nutritional absorption and metabolic disorders caused by cholestatic liver cirrhosis. Firstly, all children should undergo a clinical nutritional assessment, including anthropometric measurements and biochemical indicators (33). Due to hepatosplenomegaly, ascites, and other factors, the use of weight and BMI for nutritional assessment may be inaccurate. Consequently, short-term changes in nutritional status should be evaluated using alternative parameters such as the mid-upper arm circumference (MUAC) and triceps skinfold thickness (TSF). The MUAC can evaluate the mass of muscle and fat tissue and is relatively stable in early childhood, thus allowing for the rapid identification of children with moderate to severe malnutrition. Conversely, the TSF reflects fat status and is more sensitive for assessing malnutrition than HAZ and WAZ scores (34). In addition, all children should have their vitamin D levels monitored and be treated for vitamin D deficiency if necessary. In this study, the association between HAZ or WAZ, and the duration of liver function recovery, may have been influenced by surgical stress rather than preoperative growth trajectories. This factor was not accounted for in the present investigation, and future studies with larger sample sizes are now needed to further validate these hypotheses.

### 4.6 Clinical implications and future directions

Generally, children with BA experience growth retardation and low body weight by the time they reach end-stage liver disease. Catch-up growth typically begins around six months after transplantation, peaks at two years, and then slows, highlighting the first two years as a critical period for monitoring and intervention. The more severe the preoperative growth retardation, the faster the postoperative catch-up growth. Preoperative growth status does not exert significant effects on the postoperative recovery of liver function. To fully understand the long-term growth and development of pediatric patients, large-scale studies with a longer follow-up period are now required. In addition, future research should include more indicators, such as fat-soluble vitamins, bone density and BMI to more comprehensively evaluate the growth and development status and quality-of-life of children. Moreover, mechanistic studies integrating endocrine, nutritional, and immunological markers are needed to better elucidate determinants of growth recovery.

## 5 Conclusion

In conclusion, catch-up growth after liver transplantation in pediatric patients with biliary atresia mainly occurs within the first two years and is significantly associated with preoperative growth status and biliary complications. Children with more severe preoperative growth delay tend to exhibit more rapid postoperative growth, whereas biliary complications impair growth recovery. These findings highlight the need for early intervention and close postoperative monitoring to optimize long-term growth outcomes in this population.

## Data Availability

The raw data supporting the conclusions of this article will be made available by the authors, without undue reservation.
